# Genetic evolution and codon usage mode of SFTSV

**DOI:** 10.3389/fmicb.2025.1731572

**Published:** 2026-01-21

**Authors:** Yuhan Zhang, Li Hu, Chao Li, Xuemin Wei, Yuanyuan Shen, Hongfeng Li, Lintao Sai, Li Song, Yifei Xu

**Affiliations:** 1 Department of Health Inspection and Quarantine, School of Public Health, Cheeloo College of Medicine, Shandong University, Jinan, Shandong, China; 2 Jinan High-Tech Zone Branch of Jinan Center for Disease Control and Prevention, Jinan, Shandong, China; 3 Department of Infectious Diseases, Qilu Hospital of Shandong University, Jinan, Shandong, China; 4 Suzhou Research Institute of Shandong University, Suzhou, Jiangsu, China

**Keywords:** codon usage bias, evolution, reassortment, recombination, SFTSV

## Abstract

**Introduction:**

Severe fever with thrombocytopenia syndrome virus (SFTSV), first identified in Henan Province, China (2009), has since been detected in 26 Chinese provinces and remains a significant public health threat. Despite its spread, key aspects of its molecular evolution, particularly the genetic characterization and codon usage bias, remain understudied.

**Methods:**

In this study, we analyzed blood samples collected in 2022 from six suspected SFTS cases in Jinan, Shandong Province. This was complemented by a comprehensive analysis of whole-genome sequences obtained from the ViPR and GenBank databases, updated through July 2023. Phylogenetic analysis was employed for genotype classification. Potential recombination events were identified using RDP4. Codon usage patterns were investigated through multiple analyses, including ENC-plot, parity rule 2 (PR2) analysis, and neutral evolution analysis.

**Results:**

Phylogenetic analysis revealed that the detected viral strains belonged to genotypes C3 and C1. Recombination analysis identified 99 potential recombination events among 89 viral strains. Codon usage analyses demonstrated a weak codon usage bias in SFTSV. Further evaluation indicated that both natural selection and mutational pressure shape its codon usage patterns, with natural selection being the predominant force in four SFTSV genes.

**Discussion:**

Our findings highlight the expanding evolutionary diversity of SFTSV, evidenced by recombination events and distinct genotypes. The elucidation of its codon usage characteristics, primarily driven by natural selection, provides critical insights for advancing SFTSV surveillance, improving disease control strategies, and informing targeted vaccine development.

## Introduction

The severe fever with thrombocytopenia syndrome virus (SFTSV) is a novel bunyavirus first identified in 2009. It is a single-stranded, negative-sense RNA virus with a segmented genome consisting of three parts: the L (6,391 bp), M (3,397 bp), and S (1,760 bp) segments. SFTSV causes severe fever with thrombocytopenia syndrome (SFTS), a disease characterized by fever, thrombocytopenia, and leukopenia. In China, SFTS incidence has risen annually, with cases reported in 26 provinces and a case fatality rate ranging from 5.1 to 31%, posing a significant public health threat.

Recombination and reassortment events contribute to the genetic and genotypic diversity of SFTSV, influencing its transmissibility and pathogenicity. Codon usage bias in genes encoding key SFTSV proteins affects viral replication efficiency and pathogenicity. This bias arises from a non-equilibrium codon usage frequency shaped by factors such as mutational pressure, natural selection, and nucleotide composition (McMullan et al., 2012). Notably, codon usage patterns are virus-specific. For example, natural selection predominantly drives codon usage in H7N9 avian influenza viruses (Kim et al., 2013), Zika viruses (Wang et al., 2014), and H3N8 avian influenza viruses (Zhao et al., 2020), whereas mutational pressure plays a more dominant role in H1N1 (Darriba et al., 2012). Viral replication capacity, pathogenicity, and immune evasion can further shape codon preferences, facilitating host adaptation and driving viral evolution (Bahir et al., 2009).

Although extensive research has been conducted on SFTSV epidemiology and diagnostic methods, studies on its genetic characterization and molecular evolution remain limited. In particular, investigations into SFTSV codon usage bias are notably scarce. This study explores the evolutionary characteristics and codon usage patterns of SFTSV to elucidate its genetic evolutionary dynamics and host adaptation mechanisms. Furthermore, we analyze the relative contributions of mutational pressure and natural selection in shaping the virus’s synonymous codon usage bias. These findings provide valuable insights for selecting appropriate experimental animal models for drug and vaccine development, as well as for optimizing target gene selection in exogenous high-efficiency expression vectors.

## Materials and methods

### Sample collection and sequencing

This study was approved by the Medical Ethics Committee of Qilu Hospital, Shandong University (Approval No. LL2021120). All participants provided written informed consent in accordance with the Declaration of Helsinki.

Blood samples were collected from six suspected SFTS patients in Jinan, Shandong Province. Following storage at −4°C for 1–2 h, the samples were centrifuged at 12,000 rpm for 10 min at 4°C. The resulting supernatants were stored at −80°C until further processing. Viral RNA was extracted using the QIAamp Viral RNA Mini Kit, and SFTSV detection was performed using a commercial nucleic acid quantitative detection kit, with Ct values ≤ 35 considered positive. Positive samples were submitted to Shanghai Berger Medical Technology Co., Ltd. for whole-genome sequencing using an Illumina platform. Sequencing data were analyzed through an in-house bioinformatics pipeline to generate SFTSV consensus sequences, with quality control parameters including genome coverage and sequencing depth. Reference-based assembly was performed using BWA-MEM alignment against the HB29 reference strain (GenBank: NC_018136.1-NC_018136.3). Consensus sequences were constructed only when meeting the following quality thresholds: ≥ 10 × coverage depth and ≥ 70% major base frequency at each position.

### Data collection

Complete genome sequences of SFTSV were retrieved from the ViPR and GenBank databases (data current as of July 2023), including associated metadata containing accession numbers, collection dates, geographic locations, sequence lengths, host information, and sample types. The dataset consisted of 1,407 L segment sequences, 1,424 M segment sequences, and 1,598 S segment sequences. The classification information of the dataset is attached in Supplementary Table 1. These sequences, along with our newly generated SFTSV sequences, were stored in FASTA format. Additionally, the HB29 reference strain (GenBank accession numbers NC_018136.1-NC_018138.1) was obtained from the RefSeq database.

### Phylogenetic analyses

Phylogenetic analysis was conducted on three datasets, with nucleotide substitution models selected using jModeltest (Nguyen et al., 2015). Maximum likelihood trees for the L, M, and S segments were constructed in IQ-TREE (v2.1.4) with 1,000 bootstrap replicates (Yu et al., 2017) and visualized using the R package ggtree (Martin et al., 2010). Recombination analysis was performed using Recombination Detection Program 4 (RDP4), which incorporates seven detection algorithms (RDP (Salminen et al., 1995), Bootscan (Posada and Crandall, 2001), Chimera (Boni et al., 2007), 3Seq (Smith, 1992), GENECONV (Wong et al., 2010), Lard, and SiScan). Sequences were considered recombinant when identified by four or more methods with statistical significance (*P* < 0.05). The sequences were compared against a reference dataset of established genotypes. Putative reassortant strains were then identified based on incongruent phylogenetic placements across the three segments. Specifically, a strain was classified as a potential reassortant, which were defined by their possession of a mosaic genome where at least two of the three segments originated from divergent genotypes. Potential reassortant strains, defined as containing two or more segments of distinct genotypes, were identified through comparative phylogenetic analysis of the L, M, and S segments.

### Selection pressure analysis of SFTSV

Genetic distances for the three viral segments were calculated using the pairwise distance function in MEGA (v11.0) with the p-distance model. Nucleotide and amino acid sequence homology analyses for the L, M, and S segments were conducted using the Alignment-Sequence Identity Matrix function in BioEdit. Additionally, we employed the Nei-Gojobori method in MEGA to estimate non-synonymous (dN) and synonymous (dS) substitution rates for all three segments.

### Analysis of SFTSV codon usage bias

The Nucleotide composition function of the CALcal website^1^ and CodonW (v1.4.4) were used to analyze several indices, including the GC content of the codon third position (GC3s), the base composition at the third codon-position (A3, U3, C3, and G3), and the codon GC content.

Relative Synonymous Codon Usage (RSCU) for the SFTSV genome was calculated using the RSCU Function of the CALcal website. The RSCU values were calculated by the following formula:


$$\mathrm{RSCU}=\frac{g_{\mathrm{ij}}}{\sum_{j}^{n_{i}}g_{\mathrm{ij}}}\,n_{i}$$

Where g_*ij*_ represents the count of the ith codon for the jth amino acid, and n_*i*_ is the total occurrence of the ith codon for that amino acid (Nakamura et al., 2024; Sharp and Li, 1986).

The effective number of codons (ENC) was computed to assess synonymous codon usage bias (Comeron and Aguadé, 1998). The ENC for each protein of the SFTSV strain was calculated using CodonW, following the formula below:


$$\mathrm{ENC}=2+\frac{9}{\bar{F_{2}}}+\frac{1}{\bar{F_{3}}}+\frac{5}{\bar{F_{4}}}+\frac{3}{\bar{F_{6}}}$$

Fi (where i = 2, 3, 4, 6) represents the average values of Fi for the i-fold simplified amino acids, defined as:


$$F_{i}=\frac{n\,\sum_{j=1}^{i}{\left(\frac{n_{j}}{n}\right)}^{2}-1}{n-1}$$

1. Where n is the total number of codon occurrences for amino acids, and nj is the total occurrence of the jth amino acid codon. The ENC ranges from 20 to 61, with lower values indicating a stronger usage bias. An ENC < 35 suggests a strong bias, whereas an ENC > 55 indicates a weak bias (Wold et al., 1987). The comparison of ENC values among different genotypes was performed using one-way ANOVA, normality was verified by Shapiro-Wilk test, and homogeneity of variance was confirmed by Levene test.

ENC-Plot was utilized to evaluate the primary factors affecting codon usage bias. Expected ENC values were calculated by the following formula:


$${\mathrm{ENC}}^{\mathrm{expected}}=2+s+\left(\frac{29}{s^{2}+{\left(1-s\right)}^{2}}\right)$$

Where s represents the GC3 content. If the points on the graph align with the standard curve, it suggests that gene evolution is primarily driven by mutational pressure. Conversely, points deviating from the curve indicate with a low ENC value, suggest that codon preference may also be influenced by additional factors, such as natural selection (Wright, 1990).

### Factors driving codon usage bias of SFTSV

To assess the relative contributions of natural selection and mutational pressure on codon usage bias, we performed Parity Rule 2 (PR2) analysis. We generated a scatter plot with A3/(A3+U3) as the x-axis and G3/(G3+C3) as the y-axis, where the central point (0.5, 0.5) represents perfect parity (A = U and G = C) according to PR2. Coordinates at this origin indicate an equilibrium state where mutational pressure and natural selection exert equal influence on codon usage.

To further evaluate the relative contributions of mutational pressure versus natural selection in shaping codon usage patterns, we performed neutrality analysis. This approach examines the relationship between GC content at first and second codon positions (GC12, y-axis) and GC content at third synonymous sites (GC3s, x-axis) through linear regression analysis. The stronger the correlation between GC12 and GC3, the closer the slope of the regression curve approaches 1, indicating that codon usage bias is primarily influenced by mutation pressure. Conversely, a smaller slope of the regression line implies a greater role of natural selection pressure in shaping codon usage preferences.

We performed principal component analysis (PCA) to identify major variation patterns in codon usage (Sueoka, 1988). The analysis incorporated relative synonymous codon usage (RSCU) values from four SFTSV genes, with each gene represented as a 59-dimensional vector corresponding to its synonymous codon RSCU values. The first two principal components were analyzed to determine the primary factors influencing codon usage bias, including both mutational pressure and natural selection forces. The differences in genotype distribution were assessed using PERMANOVA.

The Codon Adaptation Index (CAI) quantifies the similarity between viral codon usage patterns and those of the host’s cellular machinery, with values ranging from 0 to 1. Values approaching 1 indicate optimal codon adaptation and potentially higher gene expression efficiency. Conversely, the Relative Codon Deoptimization Index (RCDI) assesses the divergence between viral codon usage and host reference genes, where values near 1 suggest greater codon usage similarity between virus and host. In this study, we calculated CAI and RCDI values for four SFTSV genes (L, M, NP, and NS) across seven host species: humans (Homo sapiens), domestic cats (Felis catus), dogs (Canis lupus familiaris), sheep (Ovis aries), hedgehogs (Erinaceus europaeus), raccoons (Procyon lotor), and ticks (Ixodes scapularis). Host codon usage tables were obtained from the CoCoPUTs database (Athey et al., 2017), and all indices were computed using the CAIcal web server (Hoxie and Dennehy, 2021).

## Results

### Sample sequencing

RT-qPCR analysis yielded Ct values of 19.31, 20.08, 23.02, 28.41, 27.96, and 21.06 for the six serum samples. Subsequent sequencing using the Illumina MiSeq platform generated total read counts ranging from 9,453,765 to 38,737,189, with 32.2 to 32.9% of the reads aligning to the reference genome sequence. Among the six samples, the L, M, and S fragments of SFTSV achieved 100% genome coverage at a sequencing depth of 100 × .

### Phylogenetic analysis

Using jModelTest, we identified the GTR+G nucleotide substitution model as the optimal fit for all three SFTSV gene fragments. Among the six strains isolated from SFTS patient serum samples in this study, five (SD2023–1, SD2023–2, SD2023–3, SD2023–4, and SD2023–5) were classified as clade C3, while SD2023–6 belong to clade C1 (Supplementary Figures 1–3). Phylogenetic analysis revealed that SFTSV strains segregated into nine clades (C1-C6 and J1-J3; Supplementary Figure 4). Clades C2 and C4 contained the highest number of sequences, whereas clades J1 and J2 exhibited the broadest host diversity, encompassing human, cat, tick, dog, and hedgehog isolates. Geographically, SFTSV sequences from China predominantly clustered within the C lineage, while those from Japan and Korea primarily belong to the J lineage, with J1 as the dominant genotype-a group that also included certain strains from Zhejiang and Henan, China.

### Genomic recombination and ressortment analysis

As reported previously (Zhang et al., 2024), we detected 99 potential recombination events among 89 strains using RDP4 software (Supplementary Table 2). Recombination events were more prevalent in human-derived samples (75/89) than in tick and livestock samples (10/89). Among the newly sequenced samples in this study, only the L fragment of the viral strain SD3 exhibited recombination. However, no reassortant strains were identified in the analyzed samples (Supplementary Figure 5; Ding et al., 2013; Lv et al., 2017; Yun et al., 2013; Zu et al., 2022).

### Gene homology and genetic distance analysis

Comparative sequence analysis with the reference strain revealed nucleotide and amino acid homology across all four open reading frames (ORFs). The results are presented in Table 1. The SFTSV strains demonstrated high sequence conservation, with nucleotide homologies of 97.9–100% (L segment), 87.8–100% (M segment), and 89.6–100% (S segment). At the amino acid level, homologies were 97.9–100% for RdRp, 92.7–100% for GP, 90.7–100% for NS, and 86.9–100% for NP (Supplementary Table 3). Genetic distances calculations showed the following values: 0.005 (range: 0.00–0.02) for the L segment, 0.015 (0.00–0.06) for the M segment, and 0.047 (0.00–0.11) for the S segment (Supplementary Table 4). Analysis of the six newly sequenced strains compared to the reference strain revealed amino acid homologies of 95.2–100% across all four proteins, with NP showing complete conservation (100%). These findings indicate that the NS gene displays significantly lower nucleotide and amino acid conservation compared to the RdRp and GP genes, consistent with its greater genetic distance relative to the L and M segments.

**TABLE 1:** The nucleotide and amino acid sequence homology of the ORF between the new obtained strains and the reference strain (HB29/China/2010).

Strain	L (RdRp)		M (GP)		S (NP)		S (NSs)	
	NT	AA	NT	AA	NT	AA	NT	AA
SD1	99.6%	99.9%	99.4%	99.7%	99.7%	100%	99.4%	99.6%
SD2	99.6%	99.9%	99.3%	99.3%	99.4%	100%	99.4%	99.6%
SD3	98.9%	99.6%	99.3%	99.6%	99.4%	99.5%	99.6%	99.6%
SD4	98.6%	99.6%	98.5%	99.0%	99.4%	100%	98.1%	97.9%
SD5	99.6%	99.9%	99.5%	99.6%	99.7%	100%	99.4%	99.6%
SD6	95.7%	99.1%	95.5%	98.0%	96.5%	100%	95.2%	97.6%

### Selection pressure and mutation analysis

Global dN/dS analysis of the four SFTSV-encoded proteins was performed using MEGA 11.0 (Table 2). The analysis revealed dN/dS ratios of 0.06 for RdRp, 0.13 for GP, 0.02 for NP, and 0.18 for NS, with all values significantly below 1. These findings suggest that all four proteins have undergone strong purifying selection throughout evolution. Notably, the NSs protein exhibited the highest dN/dS value, while NP showed the lowest, indicating that NSs has been subject to relatively weaker purifying selection compared to the other proteins.

**TABLE 2:** Genes dN/dS ratios of SFTSV protein.

Protein	dN/dS
RdRp	0.06
Gp	0.13
Np	0.02
NSs	0.18

A comprehensive analysis revealed numerous recurrent mutations across RdRp, GP, NSs, and NP proteins. GP contained the highest number of mutations (70), followed by RdRp (65), NSs (26), and NP (3) (Supplementary Table 5). Among these, 27 were genotype-specific, and 9 were associated with SFTS mortality. We also identified novel mutation sites—18 in RdRp, 19 in GP, 9 in NS, and 1 in NP—highlighting previously unrecognized yet potentially significant mutational sites.

Sequence analysis further identified two conserved mutations present in all six specimens: R962S in the M segment (located within GP) and L197M in the NS segment (within NS). The NP segment exhibited the fewest amino acid mutations, with only the SD3 strain showing a V237A substitution. Additionally, strains SD4 and SD6 shared three identical mutations in the L segment (RdRp): K835R, T1038S, and A1433T. These conserved and co-occurring mutations suggest potential roles in viral adaptation or functional modulation.

### Codon usage bias among SFTSV genes

**FIGURE 1:**
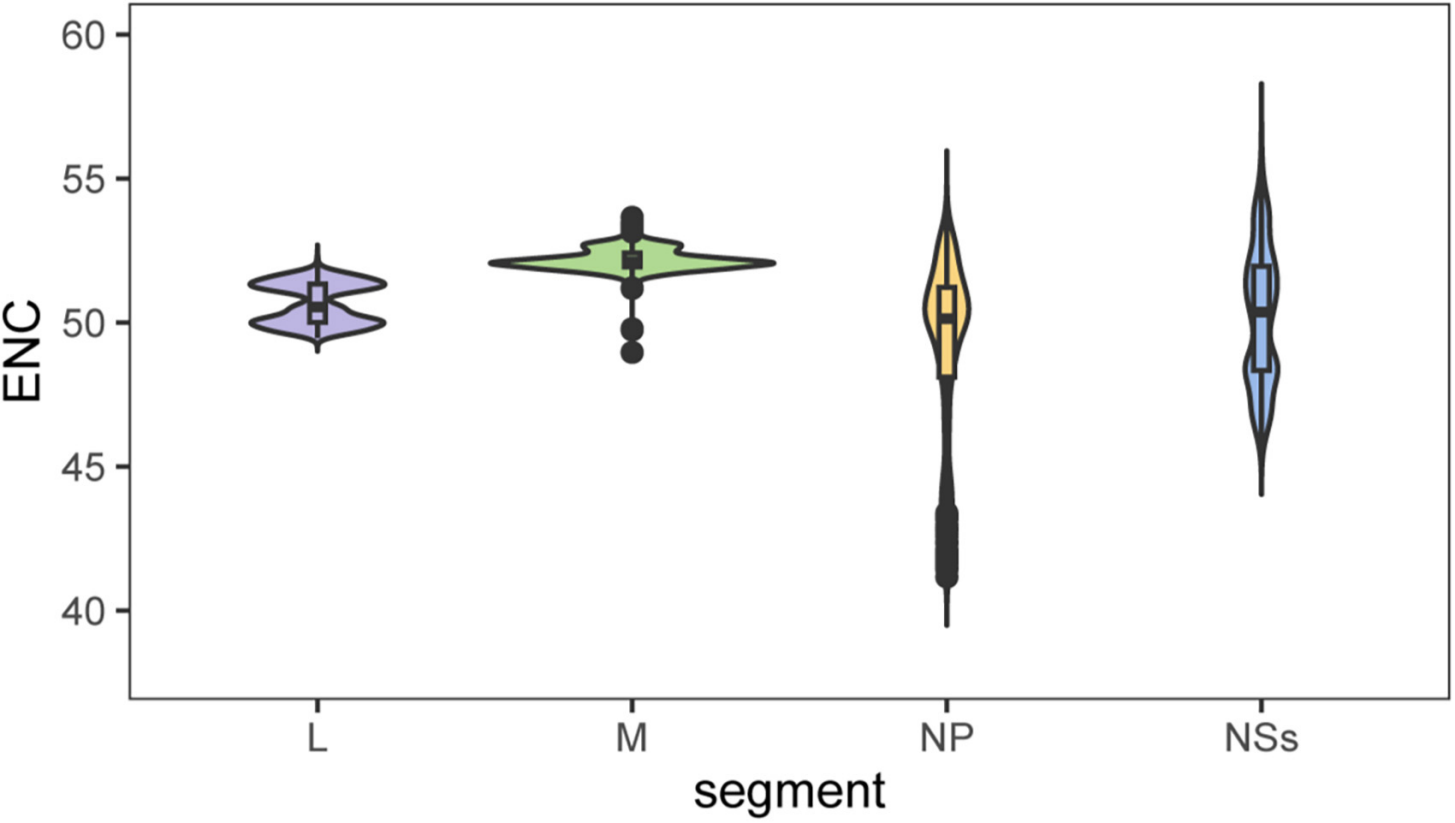
ENC analysis of the four SFTSV proteins. The vertical axis represents ENC values, while the horizontal axis represents GC3s values. The distribution range of GC3s determines whether codon preference is influenced by selective pressure and mutational pressure. The standard curve represents a scenario where codon usage bias is solely affected by mutational pressure.

**FIGURE 2:**
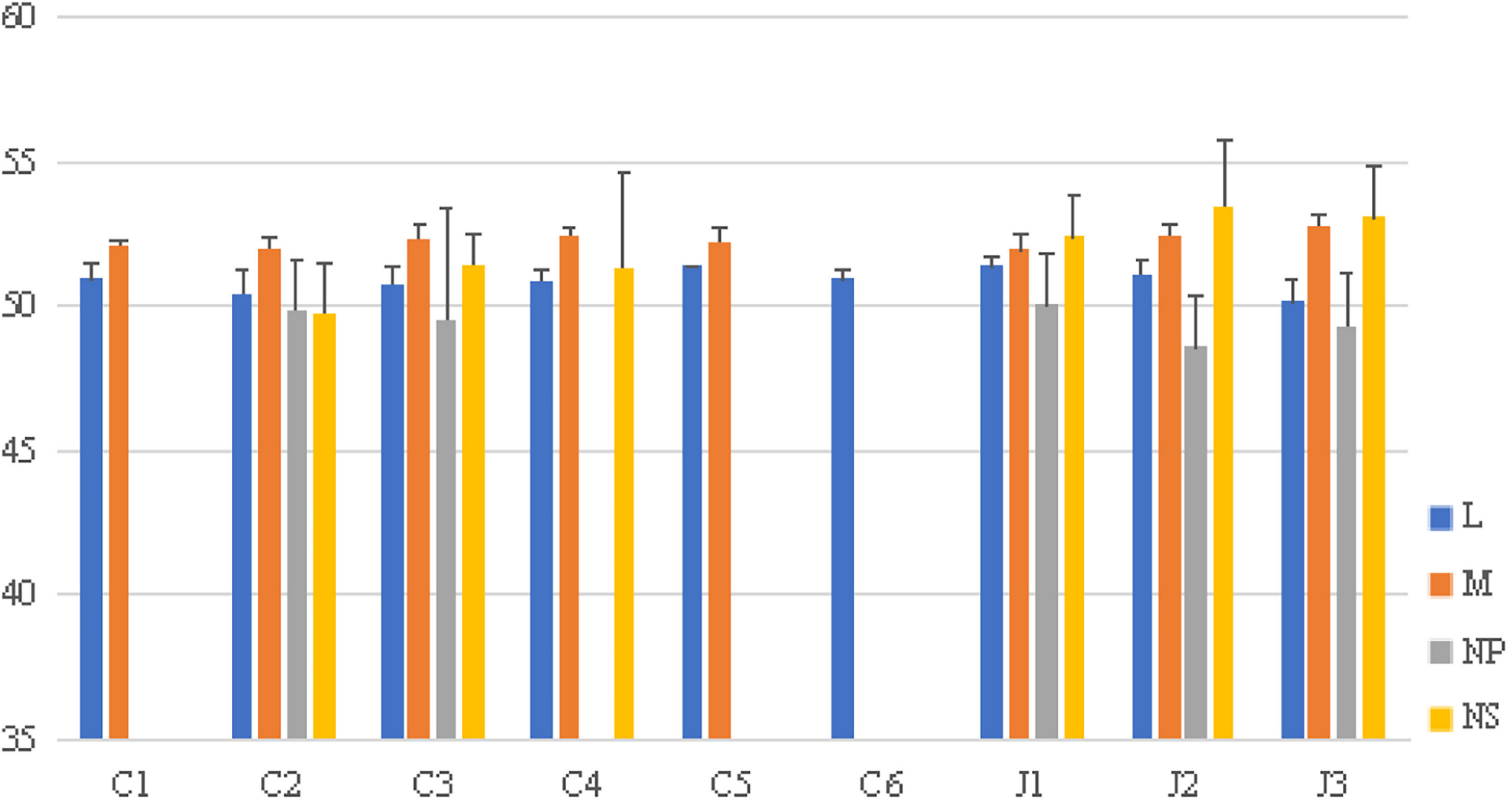
ENC values across different SFTSV genotypes. The blue, orange, gray and yellow indicate the L, M, NP and NS genes.

The effective number of codons (ENC) values were 56.67 ± 0.71 for L, 52.19 ± 0.40 for M, 49.11 ± 3.18 for NP, and 50.20 ± 2.37 for NS, all significantly exceeding the threshold of 35 (Figure 1). The L protein exhibited the highest ENC value, indicatting the weakest codon usage bias, whereas NP showed the lowest ENC. Moreover, the mean ENC values for all four proteins (L, M, NP, and NS) varied significantly among genotypes (*P* < 0.05) (Figure 2).

### Relative synonymous codon usage and principal component analysis of SFTSV

We analyzed synonymous codon usage patterns in SFTSV by calculating relative synonymous codon usage (RSCU) values for the L, M, NP, and NS genes using CAIcal. The majority of codons exhibited RSCU values between 0.6 and 1.6, while 19 codons were identified as overrepresented (RSCU > 1.6) across the four genes. These overrepresented codons included CUC (leucine, Leu), CUG (leucine, Leu), AUC (isoleucine, Ile). Noblely, overrepresented codons (RSCU > 1.6) predominantly ended in U and C, whereas underrepresented codons (RSCU < 0.6) tended to end in A and G.

This study analyzed relative synonymous codon usage (RSCU) patterns in virulent SFTSV strains across multiple host species (humans, ticks, and dogs). Among overrepresented codons, 11 showed consistent usage across all hosts, including CUG, AUC, and UCA. Notably, arginine codons AGA and AGG maintained high expression levels in all four viral genes (L, M, NP, NS) regardless of host species. In striking contrast, histidine codons (CAU and CAC) displayed distinct host—and gene-specific expression patterns, revealing divergent and often opposing codon usage trends.

**FIGURE 3:**
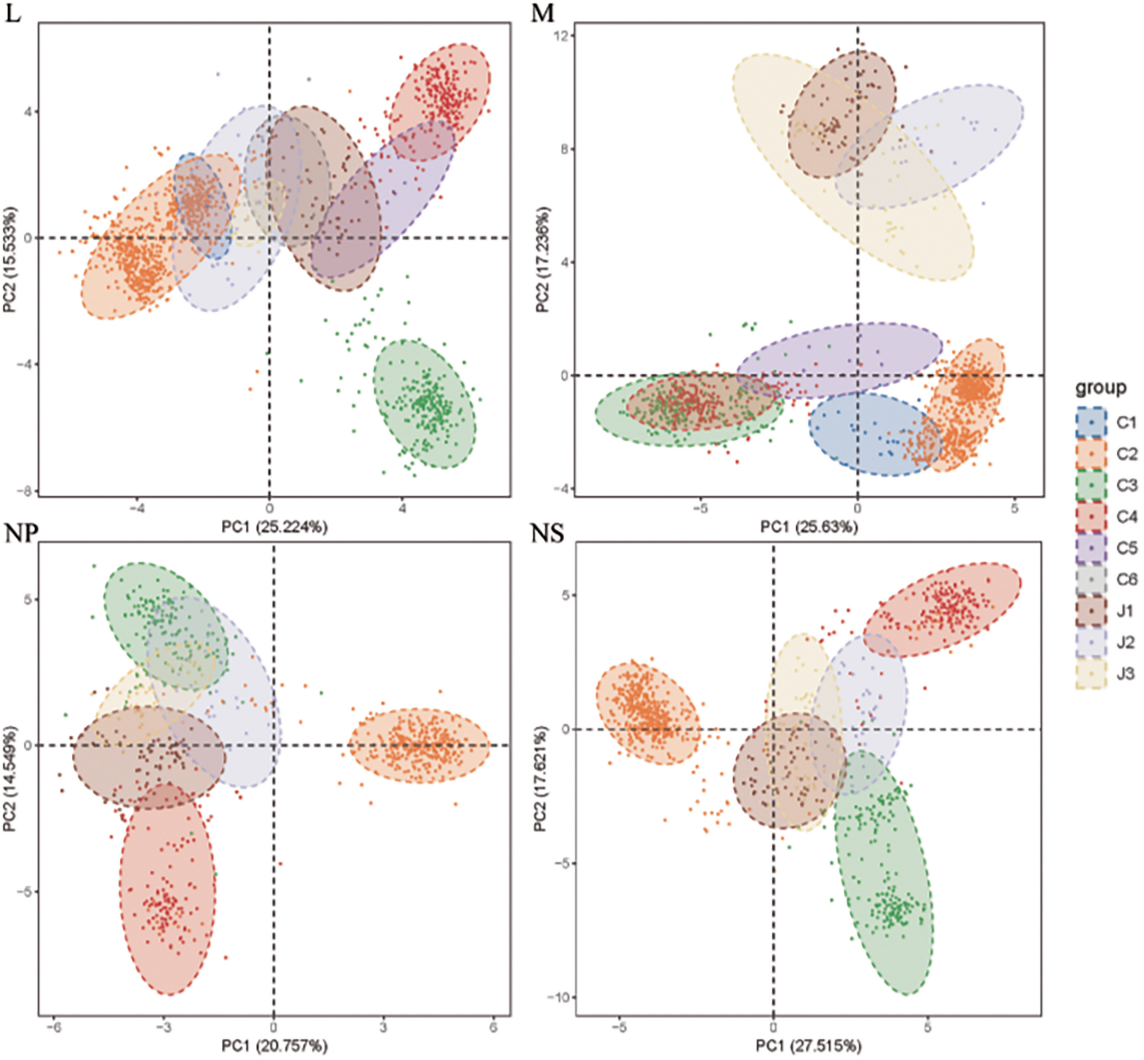
PCA results of the four SFTSV genes. The analysis was based on the RSCU values of the 59 synonymous codons. The positions of each codon were plotted on the first two-main-dimensional coordinate

We performed principal component analysis (PCA) on relative synonymous codon usage (RSCU) values of 59 codons from the four SFTSV genes, stratified by genotype (Figure 3). The analysis revealed distinct contributions of the first and second principal components (PC1 and PC2) to codon usage variation. For the L gene, PC1 and PC2 accounted for 25.2 and 15.5% of the variance, respectively (*P* < 0.001); for the M gene, the contributions were 25.6 and 17.2% (*P* < 0.001); the NP gene exhibited contributions of 20.8 and 14.5% (*P* < 0.001); while the NS gene had contributions of 27.5 and 17.6% (*P* < 0.001). PC1 emerged as the dominant factor influencing codon bias across all genes, though no single component fully explained codon usage patterns. Genotype-specific clustering along principal axes aligned with phylogenetic analysis results. Notably, J1 strains exhibited greater dispersion in clustering, reflecting higher genotypic diversity compared to other genotypes.

Both PC1 and PC2 demonstrated correlations with multiple genetic parameters, including CAI, Codon Bias Index (CBI), Frequency of Optimal Codons (Fop), ENC, GC3s, GC, GRAVY scores, and aromaticity (Aromo). Most correlation coefficients between these parameters and either PC1 or PC2 were below 0.5. These findings suggest that SFTSV’s codon usage bias arises from the combined influence of multiple evolutionary factors rather than being dominated by any single parameter.

### Factors driving codon usage bias of SFTSV genes

**FIGURE 4:**
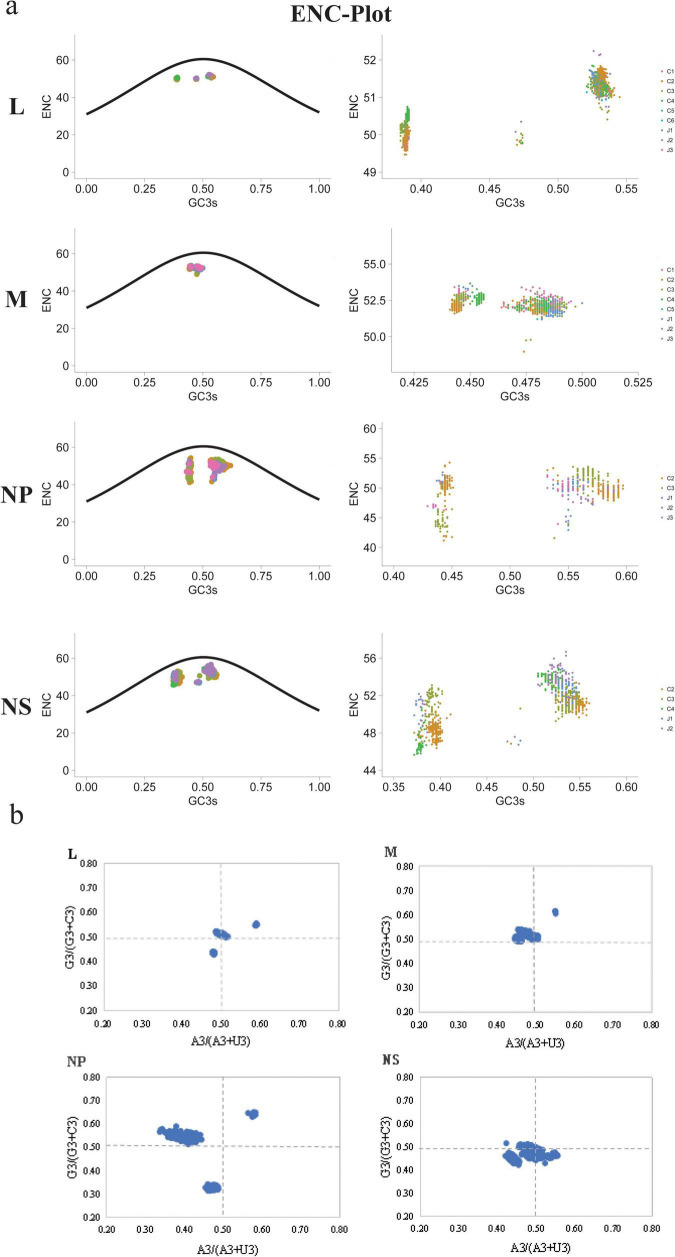
ENC-plot analysis and PR2 analysis of the four SFTSV genes. **(a)** ENC-plot analysis: the black curve represents the expected curve of SFTSV strain position. **(b)** Parity Rule 2 (PR2) analysis: points far from the origin (0.5, 0.5) indicate a bias between the influence of mutation pressure and natural selection on codon usage.

We conducted ENC-plot analysis to investigate the determinants of codon usage bias in SFTSV strains (Figure 4a). All four genes exhibited data points distributed below the standard curve, suggesting that SFTSV codon usage patterns are shaped by both natural selection and additional factors beyond mutational pressure. Notably, data points for the NS protein clustered nearer to the standard curve, implying a relatively stronger influence of mutational pressure. When analyzed by genotype, distinct clustering patterns emerged, with the NP gene displaying greater dispersion of data points compared to the other three genes.

Furthermore, we performed PR2 (Parity Rule 2) analysis to evaluate the relative contributions of mutational pressure and natural selection on codon usage patterns across the four SFTSV genes (Figure 4b). The majority of SFTSV strains plotted significantly distant from the graph’s central point (0.5, 0.5), demonstrating substantial combined effects of both mutational pressure and natural selection in shaping codon usage bias.

**FIGURE 5:**
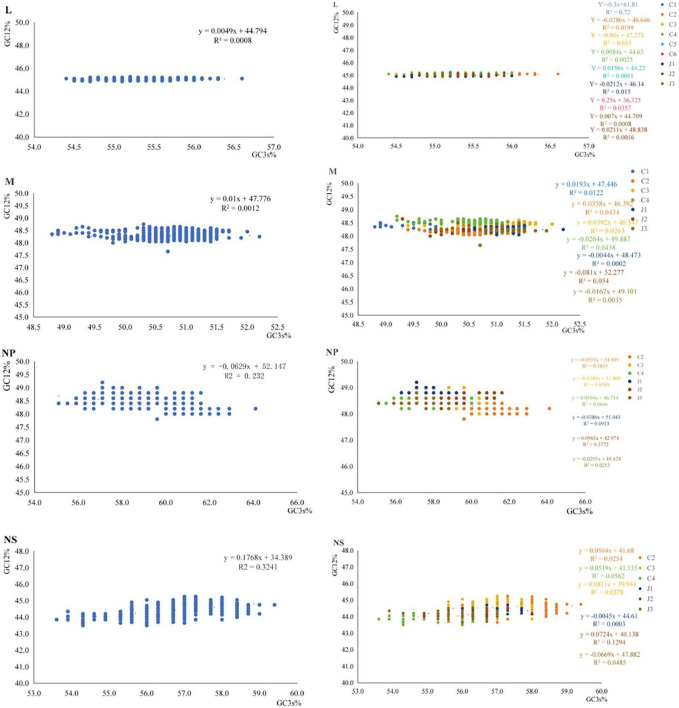
Neutral plot analysis of the four SFTSV genes. The Neutrality plot: the line represents the linear regression of GC12 on GC3. The blue points represent untyped strains. While the blue, orange, yellow, green, sky-blue, dark blue, gray and Olive drab points correspond to genotypes C2, C3, C4, J1, J2, and J3, respectively.

Through neutrality analysis (Figure 5), we quantified the relative contributions of mutational pressure and natural selection in shaping codon usage bias. Our results revealed a significant positive correlation between GC3 and GC12 content. Regression analysis demonstrated that mutational pressure accounted for only 0.49% (L gene), 1.00% (M gene), 6.29% (NP gene), and 17.68% (NS gene) of the observed variation. In contrast, natural selection dominated as the primary evolutionary force, contributing 99.51, 99.00, 93.71, and 82.32% to these respective genes. These findings clearly establish natural selection as the predominant driver of codon usage bias across all four SFTSV genes. Additionally, the NS segment exhibited the lowest dN/dS ratio, indicating it experiences weaker purifying selection compared to the other genes.

### Codon usage adaption in SFTSV

**FIGURE 6:**
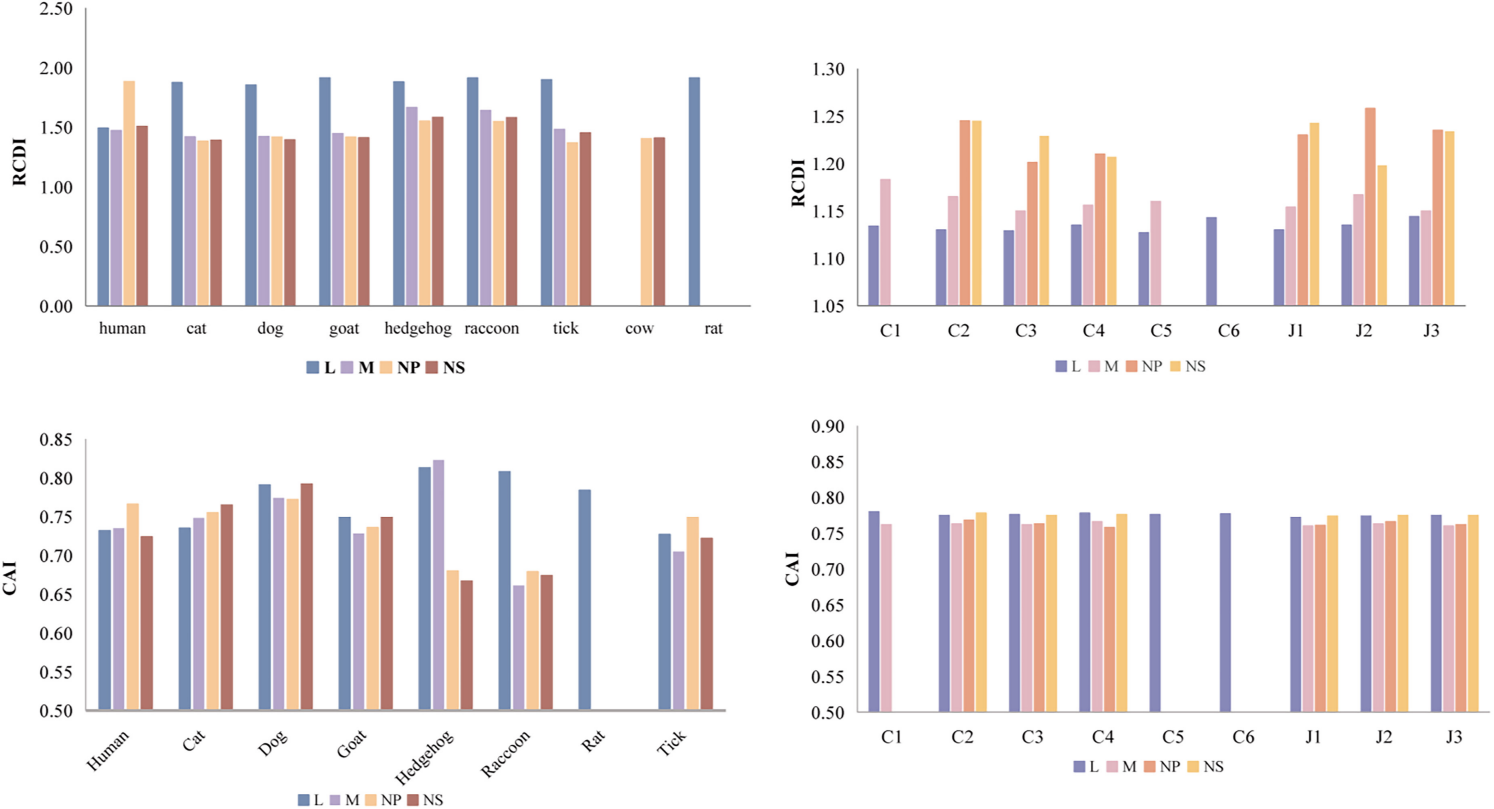
CAI and RCDI values of the four SFTSV genes. Different colors indicate the respective genes.

In this study, we calculated the Codon Adaptation Index (CAI) and Relative Codon Deoptimization Index (RCDI) values for SFTSV across seven host species (Figure 5). All analyzed genes (L, M, NP, and NS) exhibited CAI values > 0.5, indicating high translational efficiency potential. Notably, the L and M genes exhibited the highest CAI values in hedgehogs, with values of 0.813 and 0.823, respectively. In contrast, the NP and NS genes reached their maximum CAI values in dogs, at 0.772 and 0.792, suggesting higher protein synthesis efficiency in this host. Among examined hosts, the NP gene demonstrated highest adaptability to humans, while the NS gene showed the lowest. RCDI analysis revealed significantly higher values for the L gene compared to M, NP, and NS genes (Figure 6), indicating stronger codon usage similarity between the viral L gene and host genes. The remaining genes exhibited progressively lower similarity to host genes, with the following host similarity ranking: tick, cat, dog, sheep, human, raccoon, hedgehog.

## Discussion

SFTSV infection causes SFTS, presenting a significant public health challenge. As an emerging tick-borne pathogen, SFTSV infection manifests with diverse clinical presentations and carries substantial mortality risk. In the absence of approved antiviral therapies, management remains primarily supportive. Our comprehensive analysis of newly detected and publicly available SFTSV genomes identified 99 potential recombination events involving 89 viral strains. Notably, the expanding range of animal hosts (including dogs, cats, and cattle) participating in SFTSV recombination and reassortment events adds complexity to the virus’s evolutionary trajectory. Our findings further demonstrate that SFTSV exhibits relatively low codon usage bias, with natural selection serving as the predominant evolutionary force shaping codon usage patterns across all four viral genes.

In this study, we successfully amplified and sequenced complete sequences of six SFTSV strains isolated in Shandong province, while also compiling all available full-length SFTSV sequences from four affected countries. Our analysis revealed distinct geographic clustering of SFTSV genotypes: the C lineage was predominantly composed of Chinese strains, whereas the J lineage primarily contained strains from Korea and Japan (Zuo et al., 2023). Notably, we observed limited cross-geographic distribution of certain strains. Previous research by Yun et al. has proposed that migratory birds may facilitate SFTSV transmission along their flyways connecting China, Japan, and Korea (Jiang et al., 2015; Kobayashi et al., 2013). Our findings demonstrate a strong correlation between the distribution of ixodid tick populations and SFTS case occurrence. Importantly, a high prevalence of ticks was identified on migratory birds captured in SFTSV-endemic regions, suggesting these avian hosts may contribute to long-distance tick dispersal in China. This hypothesis is further supported by recent detections of SFTSV antibodies in swans and spotted doves (Darriba et al., 2012).

Recombination represents a significant mechanism driving SFTSV genetic diversity, with potential implications for viral antigenicity and virulence enhancement. Unlike positive-sense RNA viruses, negative-sense segmented RNA viruses generally demonstrate lower recombination frequencies. Our previous investigations (Gai et al., 2012; Jiang et al., 2015) have validated the occurrence of SFTSV recombination events in both human and animal host strains, with subsequent studies revealing significantly higher recombination rates in animal reservoirs (Zhang et al., 2024). Notably, recent epidemiological surveillance has detected an increasing prevalence of animal host-associated recombination events, suggesting an expanding potential for cross-species transmission and genetic diversification among circulating SFTSV strains.

Phylogenetic analyses have previously demonstrated that 6–11% of circulating SFTSV strains are reassortants (Yun et al., 2020). Our prior research identified 16 distinct reassortment patterns, which may potentially enhance viral virulence and transmission efficiency—findings with important implications for public health interventions. As a well-documented phenomenon among segmented RNA viruses, reassortment enables the generation of novel genotypes through genetic exchange between co-infecting viral strains.

Selection pressure analysis in coding genomic regions provides crucial insights for developing vaccines and antiviral therapies. Our findings demonstrate that SFTSV evolution has been predominantly shaped by purifying selection. Notably, the NS gene displayed a higher dN/dS ratio compared to other viral genes, likely reflecting its critical role in interferon antagonism—a key viral strategy for evading host immune defenses (Hofmann et al., 2013). Comparative analysis revealed that the NS protein shows the lowest sequence conservation among all viral proteins. The observed reduced sequence homology and increased genetic divergence of NSs suggest enhanced mutational flexibility, potentially facilitating viral adaptation.

Point mutations in viral genes frequently occur during transmission, often resulting in single amino acid variations within ORFs. Previous studies have demonstrated that specific amino acid substitutions can significantly alter SFTSV’s biological properties. Noda et al. reported that a mutation at position 1891 (Asn →X) in the SFTSV L segment affects polymerase activity (Noda et al., 2020). While basic amino acid substitutions (e.g., Lys) may enhance viral polymerase activity through introduced positive charges, acidic substitutions (e.g., Glu) can impair catalytic efficiency through electrostatic repulsion. Interestingly, we observed no mutations at position 1891 in our samples. Among the six analyzed strains, R962S in the M segment emerged as the most prevalent substitution, present in all newly isolated variants. Tan et al. demonstrated that Serine (Ser) at position 962 of the M segment plays a critical role in mediating cell fusion (Tani et al., 2019). Previous studies have identified seven genotype-specific amino acid mutations associated with both mortality and immune evasion, located in NP, NS, RdRp, Gn, and GP proteins—all potentially influencing viral infectivity and pathogenicity (Li A. et al., 2021; Yun et al., 2013). Our study further identified 47 novel hotspot mutations across these four proteins, warranting functional characterization. However, the precise functional mechanisms of these sites require further validation through in vitro experiments or animal models. Similarly, while the association between codon usage patterns and host fitness is suggestive, its actual impact on viral replication efficiency still needs confirmation via expression experiments or infection models. While SFTSV codon usage bias remains poorly characterized, our analysis of the four major protein-coding genes provides important insights. Genomic GC content serves as a key indicator of cross-species codon usage variation, with GC3 fluctuations potentially reflecting viral evolutionary trajectories (Sun et al., 2020). SFTSV maintains genomic GC content below 50%, indicating AU-rich composition. This finding is particularly significant as AU-rich genomes correlate with enhanced thermal stability, suggesting evolutionary adaptation to selective pressures. Codon composition (GC vs. AU-rich) significantly influences viral codon usage patterns, with preferential use of GC-ending codons (Li J. et al., 2021). Our RSCU analysis revealed that most overrepresented codons terminate in G or C. Additionally, third-position mutations occurred more frequently, reflecting their synonymous nature and consequent preservation of protein function (Koski et al., 2001). In contrast, first- and second-position mutations often cause amino acid substitutions that may alter protein structure and biological activity.

We conducted comparative analysis of Relative Synonymous Codon Usage (RSCU) patterns between SFTSV and its potential host species (humans, ticks, and domestic animals including dogs, cats, and goats). The results demonstrated that SFTSV strains from different hosts have evolved remarkably similar codon usage patterns. Principal component analysis (PCA) of RSCU values revealed that SFTSV codon bias is governed by multiple genetic determinants, with its codon usage pattern representing a balance between various evolutionary pressures.

Codon usage patterns represent a fundamental genomic feature in viruses, shaped by multiple evolutionary pressures (Butt et al., 2016). While previous studies have emphasized mutational pressure as a dominant force in RNA virus evolution, our quantitative analysis of SFTSV reveals a distinct evolutionary paradigm. Through integrated ENC-plot and PR2 analyses, we demonstrate that SFTSV’s codon usage patterns cannot be fully explained by mutational pressure alone, but rather reflect substantial selective constraints. Neutrality analysis provided compelling evidence for this disparity, with regression slopes approaching zero, indicating natural selection as the predominant driver of codon usage bias across all four SFTSV genes. These findings were consistently observed across different viral genotypes, robustly supporting our conclusion.

Natural selection drives multi-host viruses to establish an evolutionary equilibrium that facilitates survival across diverse host species (Lam et al., 2013). In this study, we evaluated codon adaptation patterns and host-specific selection pressures across seven SFTSV host species using CAI and RCDI analyses. The results revealed varying adaptation levels of SFTSV to its hosts. Notably, the L gene exhibited higher RCDI values than other genes, indicating lower similarity with viral host genes. In contrast, all four genes showed strong adaptation to tick hosts, supporting the hypothesis that ticks serve as the primary vector for SFTSV during viral latency periods-aligning with our previous findings (Luo et al., 2015).

In summary, our findings demonstrate progressive expansion of SFTSV genetic diversity, evidenced by: (1) distinct geographic distributions of viral genotypes, and (2) increasing prevalence of recombination and reassortment events. The virus maintains relatively low codon usage bias dominated by natural selection pressures, while simultaneously evolving host-optimized codon usage patterns that enhance cross-species replicative capacity. These findings provide crucial insights into viral genetic diversity and codon usage dynamics, substantially advancing our understanding of SFTSV’s evolutionary mechanisms and adaptative strategies.

## Data Availability

All data analyzed in this study were obtained from publicly accessible repositories (the Virus Pathogen Resource (ViPR) database, accessible at: https://www.bv-brc.org/ and the GenBank database, accessible at: https://www.ncbi.nlm.nih.gov/genbank/) and the corresponding accession links are provided within the article as appropriate.

## Footnotes

1
http://genomes.urv.es/CAIcal/E-CAI/

## References

[B1] Athey J. Alexaki A. Osipova E. Rostovtsev A. Santana-Quintero L. V. Katneni U. (2017). A new and updated resource for codon usage tables. *BMC Bioinform.* 18:391. 10.1186/s12859-017-1793-7 28865429 PMC5581930

[B2] Bahir I. Fromer M. Prat Y. Linial M. (2009). Viral adaptation to host: A proteome-based analysis of codon usage and amino acid preferences. *Mol. Syst. Biol.* 5:311. 10.1038/msb.2009.71 19888206 PMC2779085

[B3] Boni M. F. Posada D. Feldman M. W. (2007). An exact nonparametric method for inferring mosaic structure in sequence triplets. *Genetics* 176 1035–1047. 10.1534/genetics.106.068874 17409078 PMC1894573

[B4] Butt A. M. Nasrullah I. Qamar R. Tong Y. (2016). Evolution of codon usage in Zika virus genomes is host and vector specific. *Emerg. Microbes Infect.* 5:e107. 10.1038/emi.2016.106 27729643 PMC5117728

[B5] Comeron J. M. Aguadé M. (1998). An evaluation of measures of synonymous codon usage bias. *J. Mol. Evol.* 47 268–274. 10.1007/pl00006384 9732453

[B6] Darriba D. Taboada G. L. Doallo R. Posada D. (2012). jModelTest 2: More models, new heuristics and parallel computing. *Nat. Methods* 9:772. 10.1038/nmeth.2109 22847109 PMC4594756

[B7] Ding N. Z. Luo Z. F. Niu D. D. Ji W. Kang X. H. Cai S. S. (2013). Identification of two severe fever with thrombocytopenia syndrome virus strains originating from reassortment. *Virus Res.* 178 543–546. 10.1016/j.virusres.2013.09.017 24055465

[B8] Gai Z. Liang M. Zhang Y. Zhang S. Jin C. Wang S. W. (2012). Person-to-person transmission of severe fever with thrombocytopenia syndrome bunyavirus through blood contact. *Clin. Infect. Dis.* 54 249–252. 10.1093/cid/cir776 22095565 PMC3245727

[B9] Hofmann H. Li X. Zhang X. Liu W. Kühl A. Kaup F. (2013). Severe fever with thrombocytopenia virus glycoproteins are targeted by neutralizing antibodies and can use DC-SIGN as a receptor for pH-dependent entry into human and animal cell lines. *J. Virol.* 87 4384–4394. 10.1128/JVI.02628-12 23388721 PMC3624395

[B10] Hoxie I. Dennehy J. J. (2021). Rotavirus a genome segments show distinct segregation and codon usage patterns. *Viruses* 13:1460. 10.3390/v13081460 34452326 PMC8402926

[B11] Jiang X. L. Zhang S. Jiang M. Bi Z. Q. Liang M. F. Ding S. J. (2015). A cluster of person-to-person transmission cases caused by SFTS virus in Penglai, China. *Clin. Microbiol. Infect.* 21 274–279. 10.1016/j.cmi.2014.10.006 25687766

[B12] Kim K. H. Yi J. Kim G. Choi S. J. Jun K. I. Kim N. H. (2013). Severe fever with thrombocytopenia syndrome, South Korea, 2012. *Emerg. Infect. Dis.* 19 1892–1894. 10.3201/eid1911.130792 24206586 PMC3837670

[B13] Kobayashi Y. Kato H. Yamagishi T. Shimada T. Matsui T. Yoshikawa T. (2013). Severe fever with thrombocytopenia syndrome, Japan, 2013-2017. *Emerg. Infect. Dis.* 26 692–699. 10.3201/eid2604.191011 32186502 PMC7101122

[B14] Koski L. B. Morton R. A. Golding G. B. (2001). Codon bias and base composition are poor indicators of horizontally transferred genes. *Mol. Biol. Evol.* 18 404–412. 10.1093/oxfordjournals.molbev.a003816 11230541

[B15] Lam T. T. Y. Liu W. Bowden T. A. Cui N. Zhuang L. Liu K. (2013). Evolutionary and molecular analysis of the emergent severe fever with thrombocytopenia syndrome virus. *Epidemics* 5 1–10. 10.1016/j.epidem.2012.09.002 23438426 PMC4330987

[B16] Li A. Liu L. Wu W. Liu Y. Huang X. Li C. (2021). Molecular evolution and genetic diversity analysis of SFTS virus based on next-generation sequencing. *Biosaf. Health* 3 105–115. 10.1016/j.bsheal.2021.02.002

[B17] Li J. Li S. Yang L. Cao P. Lu J. (2021). Severe fever with thrombocytopenia syndrome virus: A highly lethal bunyavirus. *Crit. Rev. Microbiol.* 47 112–125. 10.1080/1040841X.2020.1847037 33245676

[B18] Luo L. M. Zhao L. Wen H. L. Zhang Z. T. Liu J. W. Fang L. Z. (2015). *Haemaphysalis longicornis* ticks as reservoir and vector of severe fever with thrombocytopenia syndrome virus in China. *Emerg. Infect. Dis.* 21 1770–1776. 10.3201/eid2110.150126 26402039 PMC4593435

[B19] Lv Q. Zhang H. Tian L. Zhang R. Zhang Z. Li J. (2017). Novel sub-lineages, recombinants and reassortants of severe fever with thrombocytopenia syndrome virus. *Ticks Tick Borne Dis.* 8 385–390. 10.1016/j.ttbdis.2016.12.015 28117273

[B20] Martin D. P. Lemey P. Lott M. Moulton V. Posada D. Lefeuvre P. (2010). RDP3: A flexible and fast computer program for analyzing recombination. *Bioinformatics* 26 2462–2463. 10.1093/bioinformatics/btq467 20798170 PMC2944210

[B21] McMullan L. K. Folk S. M. Kelly A. J. MacNeil A. Goldsmith C. S. Metcalfe M. G. (2012). A new phlebovirus associated with severe febrile illness in Missouri. *N. Engl. J. Med.* 367 834–841. 10.1056/NEJMoa1203378 22931317

[B22] Nakamura T. Sugeno N. Hasegawa T. Ikeda K. Yoshida S. Ishiyama S. (2024). Alpha-synuclein promotes PRMT5-mediated H4R3me2s histone methylation by interacting with the BAF complex. *FEBS J.* 291 1892–1908. 10.1111/febs.17037 38105619

[B23] Nguyen L.-T. Schmidt H. A. von Haeseler A. Minh B. Q. (2015). IQ-TREE: A fast and effective stochastic algorithm for estimating maximum-likelihood phylogenies. *Mol. Biol. Evol.* 32 268–274. 10.1093/molbev/msu300 25371430 PMC4271533

[B24] Noda K. Tsuda Y. Kozawa F. Igarashi M. Shimizu K. Arikawa J. (2020). The polarity of an amino acid at position 1891 of severe fever with thrombocytopenia syndrome virus L protein is critical for the polymerase activity. *Viruses* 13:33. 10.3390/v13010033 33375489 PMC7823514

[B25] Posada D. Crandall K. A. (2001). Evaluation of methods for detecting recombination from DNA sequences: Computer simulations. *Proc. Natl. Acad. Sci. U. S. A.* 98 13757–13762. 10.1073/pnas.241370698 11717435 PMC61114

[B26] Salminen M. O. Carr J. K. Burke D. S. McCUTCHAN F. E. (1995). Identification of breakpoints in intergenotypic recombinants of HIV type 1 by bootscanning. *AIDS Res. Hum. Retroviruses* 11 1423–1425. 10.1089/aid.1995.11.1423 8573403

[B27] Sharp P. M. Li W.-H. (1986). Codon usage in regulatory genes in *Escherichia coli* does not reflect selection for ‘rare’ codons. *Nucleic Acids Res.* 14 7737–7749. 10.1093/nar/14.19.7737 3534792 PMC311793

[B28] Smith J. M. (1992). Analyzing the mosaic structure of genes. *J. Mol. Evol.* 34 126–129. 10.1007/BF00182389 1556748

[B29] Sueoka N. (1988). Directional mutation pressure and neutral molecular evolution. *Proc. Natl. Acad. Sci. U. S. A.* 85 2653–2657. 10.1073/pnas.85.8.2653 3357886 PMC280056

[B30] Sun J. Zhao W. Wang R. Zhang W. Li G. Lu M. (2020). Analysis of the codon usage pattern of HA and NA genes of H7N9 influenza a virus. *Int. J. Mol. Sci.* 21:7129. 10.3390/ijms21197129 32992529 PMC7583936

[B31] Tani H. Kawachi K. Kimura M. Taniguchi S. Shimojima M. Fukushi S. (2019). Identification of the amino acid residue important for fusion of severe fever with thrombocytopenia syndrome virus glycoprotein. *Virology* 535 102–110. 10.1016/j.virol.2019.06.014 31299486

[B32] Wang J. Selleck P. Yu M. Ha W. Rootes C. Gales R. (2014). Novel phlebovirus with zoonotic potential isolated from ticks, Australia. *Emerg. Infect. Dis.* 20 1040–1043. 10.3201/eid2006.140003 24856477 PMC4036776

[B33] Wold S. Esbensen K. Geladi P. (1987). Principal component analysis. *Chemometr. Intell. Lab. Syst.* 2 37–52. 10.1016/0169-7439(87)80084-9

[B34] Wong E. H. Smith D. K. Rabadan R. Peiris M. Poon L. L. (2010). Codon usage bias and the evolution of influenza a viruses. Codon usage biases of influenza virus. *BMC Evol. Biol.* 10:253. 10.1186/1471-2148-10-253 20723216 PMC2933640

[B35] Wright F. (1990). The ‘effective number of codons’ used in a gene. *Gene* 87 23–29. 10.1016/0378-1119(90)90491-9 2110097

[B36] Yu G. Smith D. K. Zhu H. Guan Y. Lam T. T. (2017). Ggtree: An r package for visualization and annotation of phylogenetic trees with their covariates and other associated data. *Methods Ecol. Evol.* 8 28–36. 10.1111/2041-210x.12628

[B37] Yun M. R. Ryou J. Choi W. Lee J. Y. Park S. W. Kim D. W. (2013). Genetic diversity and evolutionary history of Korean isolates of severe fever with thrombocytopenia syndrome virus from 2013-2016. *Arch. Virol.* 165 2599–2603. 10.1007/s00705-020-04733-0 32699980 PMC7547961

[B38] Yun S. M. Park S. J. Kim Y. I. Park S. W. Yu M. A. Kwon H. I. (2020). Genetic and pathogenic diversity of severe fever with thrombocytopenia syndrome virus (SFTSV) in South Korea. *JCI Insight* 5:e129531. 10.1172/jci.insight.129531 31877113 PMC7098714

[B39] Zhang Y. Li C. Wang Y. Wei X. Xu Y. (2024). Investigation of the genetic evolutionary mechanisms of *Dabie bandavirus*. *Chin. J. Zoonoses* 40 171–178.

[B40] Zhao L. Li J. Cui X. Jia N. Wei J. Xia L. (2020). Distribution of *Haemaphysalis longicornis* and associated pathogens: Analysis of pooled data from a China field survey and global published data. *Lancet Planet. Health* 4 e320–e329. 10.1016/S2542-5196(20)30145-5 32800150

[B41] Zu Z. Lin H. Hu Y. Zheng X. Chen C. Zhao Y. (2022). The genetic evolution and codon usage pattern of severe fever with thrombocytopenia syndrome virus. *Infect. Genet. Evol.* 99:105238. 10.1016/j.meegid.2022.105238 35144005

[B42] Zuo L. L. Miao J. He D. M. Fang Z. X. Zhang X. Sun C. Y. (2023). Development and characterization of a digital CRISPR/Cas13a based assay for rapid and sensitive diagnosis of severe fever with thrombocytopenia syndrome virus. *Sens. Actuators B Chem.* 388:133789. 10.1016/j.snb.2023.133789

