# Supplementary material for: Genetic evolution and codon usage mode of SFTSV
Source: Front Microbiol. 2026 Jan 21;16:1731572. doi: 10.3389/fmicb.2025.1731572 (PMC12869193; doi:10.3389/fmicb.2025.1731572)
Supplement: Supplementary file 1 [file Table_1.docx]

Supplementary Figure1 Partial plot of phylogenetic phylogenetic tree of SFTSV sequences (L fragments)

Supplementary Figure2 Partial plot of phylogenetic phylogenetic tree of SFTSV sequences (M fragments)

Supplementary Figure3 Partial plot of phylogenetic phylogenetic tree of SFTSV sequences (S fragments)


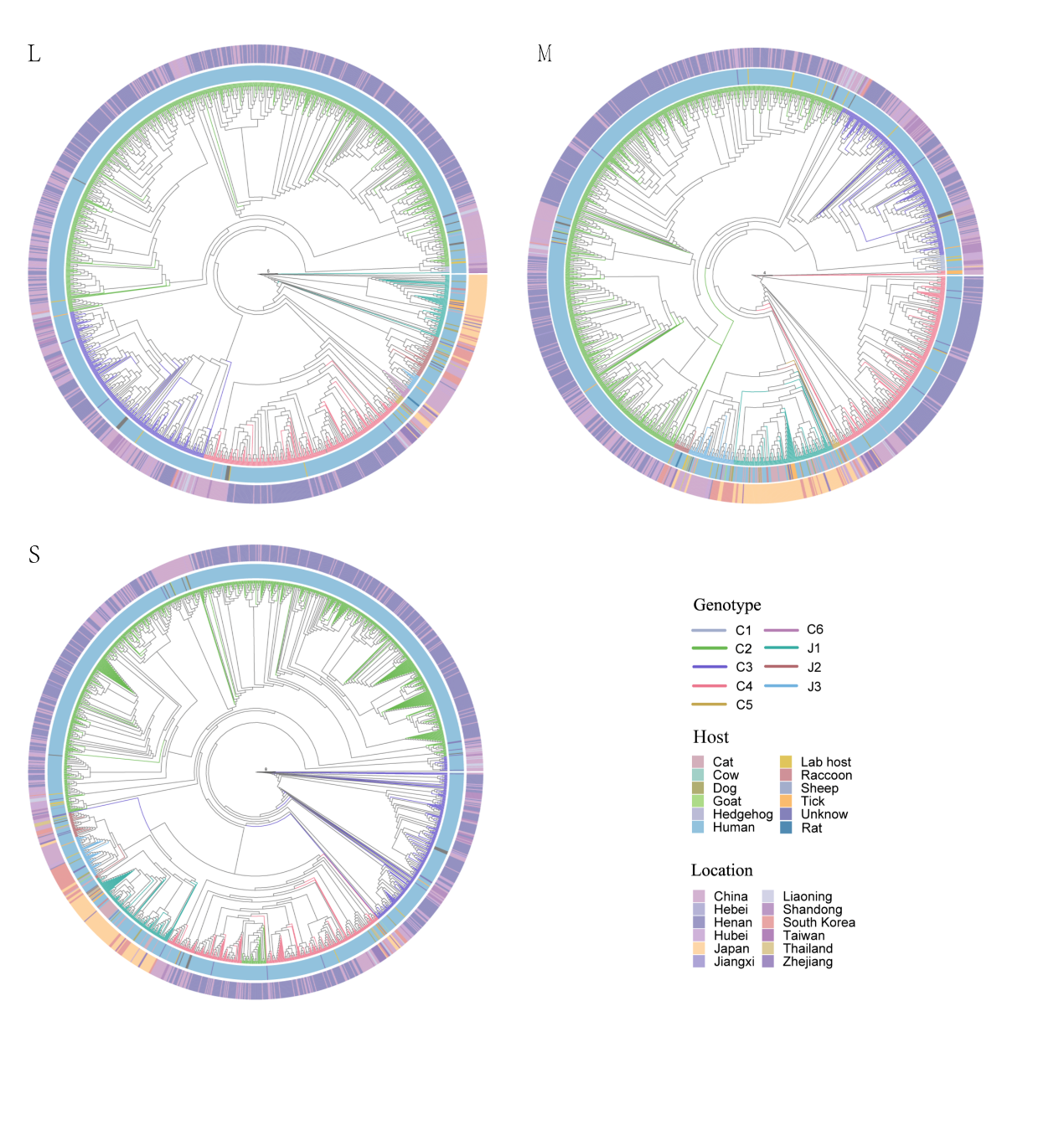


Supplementary Figure4 Phylogenetic analysis of SFTSV strains. The outer two circles of the evolutionary tree indicate the host and region of the virus, respectively.


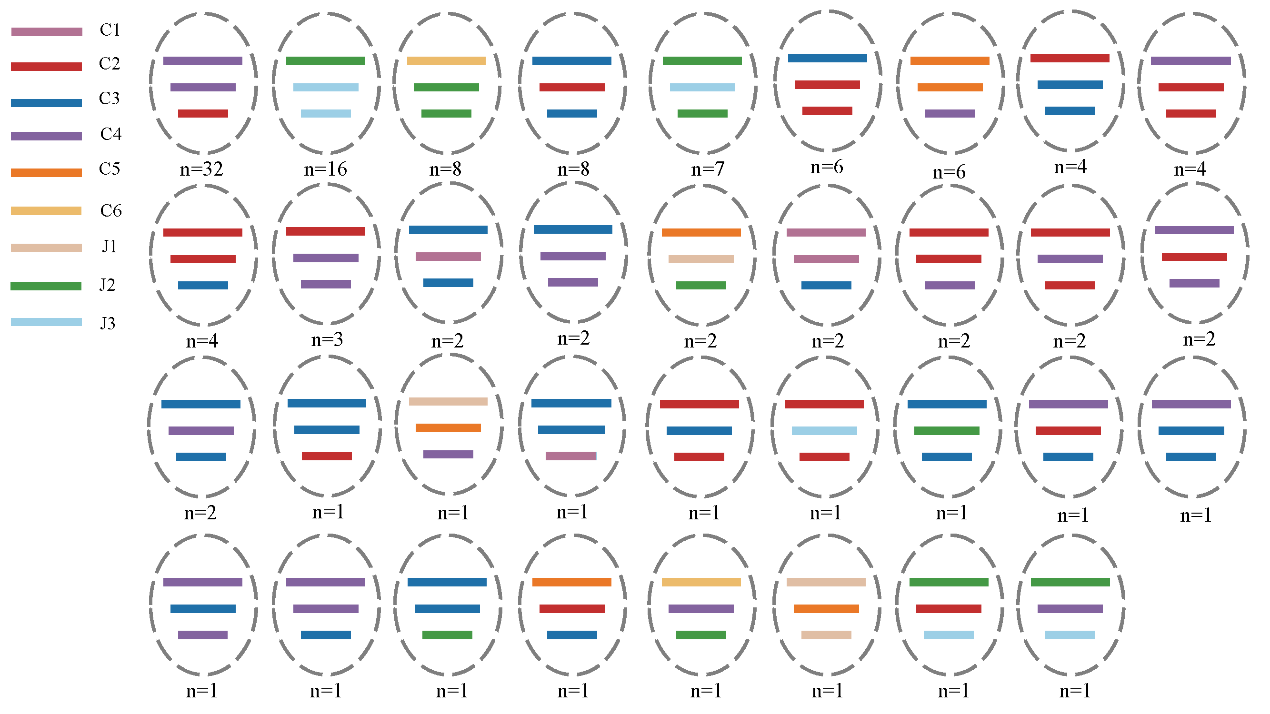


Supplementary Figure5 Genetic constellation of the 35 potential SFTSV reassortants, ‘*n’* in the figure represents the number of virulent strains.

Supplementary Table 1 Statistical Analysis of Host, Geographic, and Temporal Distribution of SFTSV Genomic Fragments (L, M, S)

|  |  | L | M | S |
| --- | --- | --- | --- | --- |
| Host | Total | 1250 | 1310 | 1460 |
|  | Human | 1193 | 1185 | 1353 |
|  | Tick | 24 | 27 | 24 |
|  | Nonhuman mammal | 21 | 77 | 51 |
|  | Lab | 12 | 21 | 12 |
|  | Unknown | 0 | 0 | 20 |
| Location | Total | 1276 | 1143 | 1141 |
|  | China | 1273 | 1141 | 1139 |
|  | Hebei | 1 | 1 | 1 |
|  | Henan | 770 | 658 | 641 |
|  | Hubei | 121 | 112 | 109 |
|  | Jilin | 1 | 0 | 1 |
|  | Jiangxi | 2 | 2 | 2 |
|  | Liaoning | 17 | 17 | 17 |
|  | Shandong | 43 | 43 | 43 |
|  | Zhejiang | 16 | 15 | 15 |
|  | Taiwan | 2 | 1 | 1 |
|  | Japan | 120 | 122 | 73 |
|  | South Korea | 61 | 44 | 36 |
| Year | Total | 1250 | 1310 | 1460 |
|  | 2010 | 3 | 3 | 3 |
|  | 2011 | 53 | 47 | 54 |
|  | 2012 | 94 | 98 | 103 |
|  | 2013 | 179 | 189 | 190 |
|  | 2014 | 164 | 159 | 241 |
|  | 2015 | 140 | 146 | 183 |
|  | 2016 | 99 | 100 | 97 |
|  | 2017 | 106 | 105 | 108 |
|  | 2018 | 124 | 127 | 121 |
|  | 2019 | 111 | 131 | 113 |
|  | 2020 | 121 | 121 | 119 |
|  | 2021 | 2 | 2 | 2 |
|  | 2022 | 6 | 9 | 6 |
|  | Unknown | 47 | 72 | 119 |

Supplementary Table 2 SFTSV recombination events detected using the RDP4 package

| virus strain | gene fragment | genotype | host | region |
| --- | --- | --- | --- | --- |
| 2011YSC60 | L^b^ | C4C2C4 | Human | Henan |
| HB2012-171 | L^b^ | C2 | Human | Hubei |
| 13-China_Henan-275 | L&M^a^ | C4 | Human | China |
| 2011YXX9 | L^b^&M&S^a^ | C3 | Human | Henan |
| HNXY_31 | L&M^a，b^ | C2 | Human | Henan |
| NB32/CHN/2013 | L&M^a，b^ | C6C4J2 | Human | China |
| SD2011-036 | L&M^a^ | C3 | Human | Shandong |
| AHZ/2020-01 | L&M^a^ | C2 | Human | China |
| SD2014-209 | L^b^ | C3 | Human | Shandong |
| 15KS15 | L&M^a^ | J2J3J3 | Human | South Korea |
| HBHG2022-12/Huanggang_Qichun/2022 | L^b^ | J2 | Human | China |
| JS2015-69 | L&Ma | J2J3J2 | Human | China |
| SD2011-038 | L&Sa | C3 | Human | Shandong |
| SDtickSFTS2018-01 | L | C3 | Tick | Shandong |
| SD3 | L | C3 | Human | Shandong |
| 16-China_Henan-598 | L | C3 | Human | Henan |
| 15KS51 | L | J2J3J3 | Human | South Korea |
| 15KS75 | L | J2 | Human | South Korea |
| 14-China_Henan-555 | L | C4C4C2 | Human | Henan |
| JS2011-46 | L | C4 | Human | China |
| 19-China_Henan-129 | L | C4 | Human | Henan |
| 17-China_Hubei-102 | L | C2 | Human | Hubei |
| 13-China_Henan-297 | L | C3 | Human | Henan |
| 19-China_Henan-137 | L | C2 | Human | Henan |
| HB2016-080 | L | J2J3J3 | Human | Hubei |
| HB2015-19 | L | C5C5C4 | Lab host | Hubei |
| 19-China_Henan-273 | L | C2 | Human | Henan |
| 19-China_Henan-264 | L | C2 | Human | Henan |
| SFTSV/Dog/1/Thailand/2022 | L | J2J3J2 | Dog | Japan |
| KAJNH2 | L | J2J3J3 | Human | South Korea |
| CB7 | L | J1 | Human | South Korea |
| 17-China_Henan-72 | L | C2 | Human | Henan |
| SPL117A | L | J1 | Human | Japan |
| TNB1590 | L | C5C5C4 | Raccoon | Japan |
| DS03/CHN/2013 | L | C5J1J2 | Human | China |
| QD7 | L | C2 | Human | China |
| HB2017-02 | L | C3J2C3 | Human | Hubei |
| 12-China_Henan-6 | M&Sa | C2 | Human | China |
| 18-China_Henan-61 | Mb | C3 | Human | Henan |
| 20-China_Henan-14 | M&Sa | J2 | Human | Henan |
| 12-China_Henan-40 | M | C3 | Human | Henan |
| 13-China_Henan-258 | M | C2 | Human | Henan |
| LNDLChina2014-51 | Mb | C3 | Human | China |
| 13-China_Henan-365 | M | C2 | Human | China |
| 14-China_Henan-236 | M | C4C4C2 | Human | China |
| 15-China_Henan-226 | M | C4C4C2 | Human | Henan |
| 18-China_Hubei-123 | M | C3 | Human | Hubei |
| 2011YSH52 | M | C4 | Human | Henan |
| 2012YSC6 | M | C2 | Human | Henan |
| HB2016-003 | M | C3 | Human | Hubei |
| HB2016-038 | M | C2C1C2 | Human | Hubei |
| HB2016-073 | M | J2J3J3 | Human | Hubei |
| IP417 | M | J3 | Cat | South Korea |
| Jilin | M | C4 | Tick | China |
| JS2011-92 | M | C2 | Human | China |
| JS2013-69 | M | C2 | Human | China |
| JS2014-16 | M | J2J3J2 | Human | China |
| JS2014-25 | M | C4 | Human | China |
| KUVL-87 | M | C5 | Cat | Japan |
| LN2010-017 | M | C3 | Human | Liaoning |
| SD2012-017 | M | C2C1C2 | Human | Shandong |
| SD2013-058 | M | C3 | Human | Shandong |
| SPL087A | M | C5C5C4 | Human | Japan |
| Ydog34 | M | C4 | Human | Japan |
| ZJZHSH-WSH/China/08/2012 | M | C4 | Human | China |
| ZJZHSH-XAM/China/06/2012 | M | J3 | Human | China |
| SPL030A | M&Sa | J1 | Human | Japan |
| 18-China_Hubei-186 | Sb | C3 | Human | Hubei |
| SDLZP07/2011 | S | C2 | Human | China |
| HB2015-39 | S | C3 | Lab host | Hubei |
| HB123/China/2011 | S | C3 | Unknown | China |
| HB3-sheep03/China/2011 | S | C3 | Sheep | China |
| NB38/CHN/2013 | S | C4C4J2 | Human | China |
| SDLZCattle01/2011 | S | C3 | Cow | China |
| KACNH | S | C3 | Human | South Korea |
| SDLZSheep01/2011 | S | C2 | Sheep | China |
| HB2015-10 | S | C2C1C2 | Lab host | Hubei |
| 13-China_Henan-182 | S | C4 | Human | China |
| 17-China_Henan-255 | S | C2 | Human | Henan |
| 14-China_Henan-84 | S | C4C4C2 | Human | Henan |
| XCQ-A112S | S | C2 | Tick | China |
| 19-China_Hubei-297 | S | C2 | Human | Hubei |
| 17-China_Hubei-125 | S | C2 | Human | China |
| AFMC 17-1 | S | J2 | Human | South Korea |
| SD2015-089 | S | C3 | Human | Shandong |
| 127S | S | C2 | Human | Henan |
| ZJZHSH-HYC/China/05/2012 | S | J3 | Human | China |
| 15MS65 | S | J2J3J3 | Human | South Korea |
| 14-China_Henan-579 | S | C4 | Human | China |

Note:a indicates that recombination events occurred simultaneously in multiple segments of a strain; b indicates that multiple recombination events occurred in a single segment.

Supplementary Table 3 SFTSV Genetic constellation of the twenty-six potential DBV reassortants

|  | L | M | S | |
| --- | --- | --- | --- | --- |
|  |  |  | NSs | NP |
| Nucleotide homology（%） | 97.9-100 | 87.8-100 | 89.6-100 | |
| Amino acid homology（%） | 97.9-100 | 92.7-100 | 90.7-100 | 86.9-100 |

Supplementary Table 4 Genetic evolutionary distance of SFTSV strains

| Gene | Genetic evolutionary distance | Average genetic distance |
| --- | --- | --- |
| L | 0.00-0.02 | 0.005 |
| M | 0.00-0.06 | 0.015 |
| S | 0.00-0.11 | 0.047 |

Supplementary Table 5 SFTSV amino acid mutation site statistics

| protein | | | locus | quantity | | protein | | locus | | quantity | | protein | | locus | | quantity | |
| --- | --- | --- | --- | --- | --- | --- | --- | --- | --- | --- | --- | --- | --- | --- | --- | --- | --- |
| RdRp | | | N2D | 98 | | Gp | | C12Y | | 6 | | NP | | S962G | | 10 | |
|  | | | N11S | 13 | |  | | F13L | | 669 | |  | | Q984K | | 20 | |
|  | | | S128T | 202 | |  | | C17S | | 2 | |  | | K988R | | 248 | |
|  | | | A140T | 35 | |  | | S18G | | 248 | |  | | T1011S | | 203 | |
|  | | | R240K | 77 | |  | | D20G | | 48 | |  | | T1011A | | 8 | |
|  | | | G243R | 57 | |  | | D20E | | 4 | |  | | I1037V | | 124 | |
|  | | | G243K | 8 | |  | | S21T | | 447 | |  | | I1037F | | 20 | |
|  | | | G243E | 7 | |  | | S21A | | 6 | |  | | V1040L | | 11 | |
|  | | | E249K | 6 | |  | | A36T | | 5 | |  | | I1041M | | 261 | |
|  | | | E251K | 34 | |  | | D37G | | 198 | |  | | F1052X | | 13 | |
|  | | | S258T | 5 | |  | | Y83F | | 123 | |  | | F1052S | | 7 | |
|  | | | S284N | 27 | |  | | K113R | | 18 | |  | | F1052Y | | 5 | |
|  | | | I299V | 288 | |  | | G114E | | 161 | |  | | F1052L | | 4 | |
|  | | | K307R | 6 | |  | | T150I | | 4 | |  | | M1053L | | 695 | |
|  | | | D310E | 9 | |  | | A169T | | 5 | |  | | M1053X | | 13 | |
|  | | | D310N | 5 | |  | | D170N | | 173 | |  | | M1055L | | 23 | |
|  | | | G314S | 154 | |  | | E217D | | 10 | |  | | M1055V | | 6 | |
|  | | | G314D | 20 | |  | | E217G | | 5 | |  | | S1056F | | 202 | |
|  | | | A317S | 9 | |  | | G218S | | 203 | |  | | F1058L | | 147 | |
|  | | | S343T | 11 | |  | | K272R | | 10 | | NS | | S7F | | 8 | |
|  | | | F352Y | 4 | |  | | T273A | | 229 | |  | | S7P | | 5 | |
|  | | | V381I | 80 | |  | | M284I | | 13 | |  | | S7T | | 4 | |
|  | | | D389G | 296 | |  | | E300D | | 10 | |  | | M16L | | 54 | |
|  | | | E397D | 55 | |  | | E300G | | 6 | |  | | M16V | | 3 | |
|  | | | K398R | 12 | |  | | L337M | | 52 | |  | | Y30F | | 53 | |
|  | | | S417T | 5 | |  | | N340S | | 199 | |  | | L33P | | 3 | |
|  | | | S417L | 3 | |  | | Q341P | | 295 | |  | | L45M | | 5 | |
|  | | | K444R | 8 | |  | | Q341S | | 60 | |  | | P66X | | 14 | |
|  | | | E448D | 40 | |  | | Q341E | | 14 | |  | | K67X | | 10 | |
|  | | | T449A | 243 | |  | | N358S | | 33 | |  | | K67E | | 4 | |
|  | | | F453Y | 6 | |  | | N358I | | 7 | |  | | N68X | | 6 | |
|  | | | E457D | 369 | |  | | K371R | | 148 | |  | | P69X | | 4 | |
|  | | | A470T | 16 | |  | | T385S | | 43 | |  | | G143S | | 235 | |
|  | | | I479V | 626 | |  | | T385M | | 4 | |  | | R144Q | | 756 | |
|  | | | A538T | 13 | |  | | K389X | | 6 | |  | | R144K | | 11 | |
|  | | | Y566F | 219 | |  | | Q394H | | 207 | |  | | R145K | | 765 | |
|  | | | I570V | 15 | |  | | K403R | | 10 | |  | | D171E | | 769 | |
|  | | | D586E | 209 | |  | | V413I | | 5 | |  | | I172T | | 65 | |
|  | | | E592D | 10 | |  | | V413A | | 4 | |  | | I172V | | 3 | |
|  | | | V634A | 5 | |  | | E417D | | 13 | |  | | H201Y | | 11 | |
|  | I651V | | | 186 | |  | | I479V | | 703 | |  | | S207P | | 151 | |
|  | D691E | | | 403 | |  | | I491M | | 470 | |  | | I223V | | 233 | |
|  | L703F | | | 56 | |  | | I491V | | 260 | |  | | I223T | | 19 | |
|  | S719N | | | 156 | |  | | I491L | | 18 | |  | | I223X | | 4 | |
|  | N828S | | | 92 | |  | | M499I | | 8 | |  | | T228A | | 4 | |
|  | R835K | | | 156 | |  | | T501A | | 257 | |  | | S233G | | 43 | |
|  | K884R | | | 39 | |  | | T501S | | 205 | |  | | S233R | | 5 | |
|  | A1008T | | | 15 | |  | | T501I | | 5 | |  | | D239E | | 56 | |
|  | S1038T | | | 156 | |  | | M506V | | 214 | |  | | D239N | | 5 | |
|  | S1045T | | | 20 | |  | | N524Y | | 4 | |  | | Q245H | | 160 | |
|  | D1061E | | | 680 | |  | | R525G | | 230 | |  | | Y249H | | 223 | |
|  | K1073E | | | 13 | |  | | Q529H | | 18 | |  | | N276D | | 233 | |
|  | S1116N | | | 74 | |  | | Q529R | | 5 | |  | | R281K | | 52 | |
|  | S1116C | | | 40 | |  | | M536I | | 16 | |  | | R281G | | 4 | |
|  | I1199V | | | 62 | |  | | T553A | | 10 | |  | | A285V | | 58 | |
|  | T1208S | | | 12 | |  | | I557M | | 27 | |  | | P289L | | 765 | |
|  | N1353S | | | 58 | |  | | I557V | | 19 | | NP | | K52R | | 235 | |
|  | T1433A | | | 308 | |  | | S562G | | 702 | |  | | R95K | | 21 | |
|  | I1447V | | | 228 | |  | | R577K | | 142 | |  | | R95T | | 1 | |
|  | T1485A | | | 11 | |  | | V587I | | 147 | |  | |  | |  | |
|  | I1500M | | | 8 | |  | | K619R | | 175 | |  | |  | |  | |
|  | I1651M | | | 53 | |  | | K621R | | 16 | |  | |  | |  | |
|  | I1659M | | | 18 | |  | | P662S | | 194 | |  | |  | |  | |
|  | R1684K | | | 161 | |  | | A727T | | 5 | |  | |  | |  | |
|  | V1717I | | | 139 | |  | | I780M | | 22 | |  | |  | |  | |
|  | K1722R | | | 7 | |  | | I818X | | 14 | |  | |  | |  | |
|  | E1749G | | | 5 | |  | | K819X | | 11 | |  | |  | |  | |
|  | K1825R | | | 56 | |  | | K926R | | 63 | |  | |  | |  | |
|  | V1837I | | | 63 | |  | | S934N | | 19 | |  | |  | |  | |
|  | K1906R | | | 40 | |  | | T960S | | 260 | |  | |  | |  | |
|  | R1913K | | | 12 | |  | | T960I | | 146 | |  | |  | |  | |

Note:Mutation loci not reported in red font; positive selection pressure loci in pink background; mutation loci associated with SFTS mortality in green background; genotype-specific mutation loci in yellow background.
